# Supplementary material for: Comparative Safety Analysis of Empagliflozin in Type 2 Diabetes Mellitus Patients with Chronic Kidney Disease versus Normal Kidney Function: A Nationwide Cohort Study in Korea
Source: Pharmaceutics. 2023 Sep 27;15(10):2394. doi: 10.3390/pharmaceutics15102394 (PMC10610004; doi:10.3390/pharmaceutics15102394)
Supplement: Supplementary file 1 [file pharmaceutics-15-02394-s001.zip › pharmaceutics-2578437-supplementary.pdf]

Table S1. Operational definitions of outcomes

	Operational Definition
**Myocardial infarction**	Myocardial infarction (I21) with procedure code
**Hospitalization for unstable angina**	Unstable angina (I20) with hospitalization
**Coronary revascularization procedure**	Procedure code [Coronary angiography (HA670), aortocoronary venous bypass graft angiography (HA680, HA681, HA682), percutaneous transluminal coronary angioplasty (M6551, M6552), percutaneous transcatheter placement of intracoronary stent (M6561, M6562, M6563, M6564), percutaneous transluminal coronary atherectomy (M6571, M6572), mechanical thrombectomy (M6633), percutaneous thrombus removal (M6634), vascular bypass operation (O1641, O1642, O1647, OA641, OA642, OA647)]
**Stroke**	Cerebrovascular diseases (I60-I64) with procedure code
**Transient ischemic attack**	Transient ischemic attack (G45)
**Hospitalization for heart failure**	Heart failure (I50)<br>Left ventricular failure (I50.1)<br>Left ventricular failure (I50.1)<br>Cardiogenic shock (R57.0)<br>Pulmonary edema (J81)<br>Systolic (congestive) heart failure (I50.2)
**Hypoglycemic adverse event**	Type 2 diabetes mellitus with hypoglycemia (E11.63), other specified diabetes mellitus with hypoglycemia (E13.64), nondiabetic hypoglycemic coma (E15),<br>drug-induced hypoglycemia without coma (E16.0), other hypoglycemia (E16.1),<br>hypoglycemia, unspecified (E16.2)
**Urinary tract infection**	Urinary tract infection, site not specified (N39.0)<br>Cystitis (N30)<br>Abnormal findings on microbiological examination of urine (R82.7)<br>Acute pyelonephritis (N10), Chronic tubulo-interstitial nephritis (N11), Tubulo-interstitial nephritis, not specified (N12)<br>Renal and perinephric abscess (N15.1) with any of above codes
**Genital tract infection**	Candida balanitis(B37.41)<br>Balanoposthitis (N48.1)<br>Cellulitis of corpus cavernosum and penis (N48.21)<br>Epididymitis and orchitis (N45)<br>Other inflammatory disorders of penis (N48.2)<br>Acute prostatitis (N41.0)<br>Chronic prostatitis (N41.1)<br>Other inflammatory diseases of prostate (N41.8)<br>Inflammatory disease of prostate, unspecified (N41.9)<br>Abscess of prostate (N41.2)<br>Inflammatory diseases of prostate (N41)<br>Acute vaginitis (N76.0)<br>Subacute and chronic vaginitis (N76.1)<br>Acute vulvitis (N76.2)<br>Subacute and chronic vulvitis (N76.3)<br>Other specified inflammation of vagina and vulva (N76.8)<br>Vaginitis, vulvitis and vulvovaginitis in diseases classified elsewhere (N77.1)<br>Vulval abscess (N76.4)<br>candida of vulva and vagina (B37.3)<br>vaginitis and vulvovaginitis (N77.1, N76.0-N76.3)<br>Candidiasis of other urogenital sites (B37.4)

	Candida of other urogenital sites (B37.48)
**Volume depletion**	Hypotension (I95.9)
	Syncope and collapse (R55)
	Dehydration (E86.0), Volume depletion, unspecified (E86.8)
	Orthostatic hypotension (I95.1)
	Nonspecific low blood-pressure reading (R03.1)
	Hypovolemia (E86.1)
**Acute kidney injury**	Acute kidney failure (N17)
**Diabetic ketoacidosis**	Diabetic ketoacidosis (E11.1, E13.1, E14.1)
**Thromboembolic event**	Other venous embolism and thrombosis (I82)
	Pulmonary embolism (I26)
	Phlebitis and thrombophlebitis (I80)
	Other retinal vein occlusions (H34.8)
	Phlebitis and thrombophlebitis, superficial (I80.0)
	Complication of vein following a procedure (T81.7)
	Vascular disorders of intestine (K55)
	Postthrombotic syndrome (I87.0)
**Bone fracture**	Fracture of sternum (S22.2)
	Fracture of rib (S22.3)
	Multiple fractures of ribs (S22.4)
	Foot Fracture (S92)
	Fracture of upper end of humerus (S42.2)
	Fracture of shaft of humerus (S42.3)
	Fracture of lower end of humerus (S42.4)
	Fracture of medial malleolus (S82.5)
	Fracture of lateral malleolus (S82.6)
	Pathological fracture, not elsewhere classified (M84.4)
	Fracture of sacrum (S32.1)
	Fracture of ilium (S32.3)
	Fracture of other and unspecified parts of lumbar spine and pelvis (S32.8)
	Fracture of ischium (S32.82)
	Fracture of upper end of radius (S52.1)
	Fracture of shaft of radius (S52.3)
	Fracture of lower end of radius (S52.5)
	Fracture of tooth (S02.5)
	Fracture of navicular [scaphoid] bone of hand (S62.0)
	Fracture of other carpal bone(s) (S62.1)
	Fracture of thumb (S62.5)
	Fracture of other finger (S62.6)
	Multiple fracture of fingers (S62.7)
	Fracture of upper end of tibia (S82.1)
	Fracture of shaft of tibia (S82.2)
	Fracture of lower end of tibia (S82.3)
	Fracture of skull and facial bones (S02, excluding S02.5, S02.6)
	Fracture of first metacarpal bone (S62.2)
	Fracture of other metacarpal bone (S62.3)
	Multiple fracture of metacarpal bones (S62.4)
	Fracture of other and unspecified parts of wrist and hand (S62.8)
	Collapsed vertebra (M48.5)
	Fracture of head and neck of femur (S72.0)
	Fracture of femur (S72)
	Fracture of fibula alone (S82.4)
	Fracture of other and unspecified parts of lumbar spine and pelvis (S32.8)
	Fracture of acetabulum (S32.4)

	Fracture of lumbar vertebra (S32.0)
	Osteoporosis with pathological fracture (M80)
	Fracture of patella (S82.0)
	Fracture of clavicle (S42.0)
	Multiple fracture of forearm (S52.7)
	Fracture of other parts of forearm (S52.8)
	Unspecified fracture of forearm (S52.9)
	Fracture of mandible (S02.6)
	Fracture of upper end of ulna (S52.0)
	Fracture of shaft of ulna (S52.2)
	Fracture of shafts of both ulna and radius (S52.4)
	Fracture of lower end of both ulna and radius (S52.6)
	Fracture of coccyx (S32.2)
	Other fractures of lower leg (S82.8)
	Unspecified fracture of lower leg (S82.9)
	Fracture of bone following insertion of orthopaedic implant, joint prosthesis, or bone plate (M96.6)
	Fracture of pubis (S32.5)
	Fracture of cervical vertebra and other parts of neck (S12)
	Fracture of scapula (S42.1)
	Fracture of base of skull (S02.1)
	Fracture of thoracic vertebra (S22.0)
	Included in each fracture code

Table S2. Baseline characteristics of patients with chronic kidney disease before and after match

Variables	Pre-Match Sitagliptin N= 20,136	Pre-Match Empagliflozin N= 6,211	STD	Post-Match Sitagliptin N= 6,170	Post-Match Empagliflozin N= 6,170	STD
Sex, male	11632 (57.8)	3478 (56)	-0.09	3534 (57.3)	3464 (56.1)	-0.02
Age, year	56.8 ± 12.7	50.8 ± 12.2	-0.48	50.8 ± 12.8	50.9 ± 12.1	0.01
Insurance type						
Normal	18730 (93.0)	5886 (94.8)	0.07	5842 (94.7)	5846 (94.8)	0.004
Medicaid	1313 (6.5)	305 (4.9)		307 (5)	304 (4.9)	
No charge	93 (0.5)	20 (0.3)		21 (0.3)	20 (0.3)	
Number of Inpatient visit	1.1 ± 2.6	0.5 ± 1.2	-0.3	0.5 ± 1.3	0.5 ± 1.3	0.003
Number of outpatient visit	38.5 ± 40.7	25.5 ± 23.9	-0.27	29.0 ± 28.4	29.0 ± 28.4	0.002
Index year						
2016	6060 (30.1)	1565 (25.2)	0.13	1545 (25)	1564 (25.4)	0.02
2017	9915 (49.2)	3078 (49.6)		3106 (50.3)	3057 (49.6)	
2018	4161 (20.7)	1568 (25.3)		1519 (24.6)	1549 (25.1)	
Charlson comorbidity index						
0	10 (0.1)	1 (0)	0.08	2 (0)	1 (0)	0.02
1	1099 (5.5)	413 (6.7)		416 (6.7)	408 (6.6)	
2	1428 (7.1)	532 (8.6)		503 (8.2)	523 (8.5)	
3	17599 (87.4)	5265 (84.8)		5249 (85.1)	5238 (84.9)	
CV risk factor						
CAD	7170 (35.6)	2067 (33.3)	-0.05	1946 (31.5)	2054 (33.3)	0.04
Multi vessel CAD	3797 (18.9)	1137 (18.3)	-0.01	1080 (17.5)	1126 (18.3)	0.02
MI	389 (1.9)	97 (1.6)	-0.03	110 (1.8)	95 (1.5)	-0.02
CABG	1752 (8.7)	433 (7.0)	-0.06	444 (7.2)	430 (7)	-0.01
Stroke	1103 (5.5)	181 (2.9)	-0.13	190 (3.1)	181 (2.9)	-0.01
PAD	1172 (5.8)	149 (2.4)	-0.17	170 (2.8)	148 (2.4)	-0.02
DM circulation	3370 (16.7)	1092 (17.6)	0.02	1078 (17.5)	1083 (17.6)	0.002
DM foot	7 (0.0)	0 (0.0)	-0.03	0 (0.0)	0 (0.0)	0
DM nephropathy	18402 (91.4)	5868 (94.5)	0.12	5789 (93.8)	5828 (94.5)	0.03
DM neuropathy	3970 (19.7)	1096 (17.7)	-0.05	1111 (18)	1089 (17.7)	-0.01
DM other Complications	14396 (71.5)	4106 (66.1)	-0.11	4101 (66.5)	4085 (66.2)	-0.01
Hyperglycemia	841 (4.2)	140 (2.3)	-0.11	126 (2)	140 (2.3)	0.02
Comorbidities						
Hypertension	14595 (72.5)	4359 (70.2)	-0.05	4312 (69.9)	4330 (70.2)	0.006
Edema	3017 (15)	704 (11.3)	-0.11	704 (11.4)	696 (11.3)	-0.004
Kidney stone	441 (2.2)	123 (2.0)	-0.01	120 (1.9)	123 (2.0)	0.004
Osteoarthritis	7062 (35.1)	1813 (29.2)	-0.13	1832 (29.7)	1808 (29.3)	-0.01
Other arthritis	6679 (33.2)	1707 (27.5)	-0.12	1672 (27.1)	1698 (27.5)	0.01
PUD	5921 (29.4)	1609 (25.9)	-0.08	1619 (26.2)	1596 (25.9)	-0.01
Pancreatitis	414 (2.1)	125 (2)	-0.003	126 (2)	124 (2)	-0.002
UC	35 (0.2)	9 (0.1)	-0.007	12 (0.2)	9 (0.2)	-0.01

Crohn	8 (0)	4 (0.1)	0.00	3 (0.1)	4 (0.1)	0.000
Asthma	3227 (16)	910 (14.7)	-0.04	946 (15.3)	899 (14.6)	-0.02
COPD	842 (4.2)	166 (2.7)	-0.08	182 (3)	164 (2.7)	-0.02
Bladder stone	19 (0.1)	3 (0.1)	-0.02	2 (0)	3 (0.1)	0.01
Dementia	2626 (13)	527 (8.5)	-0.15	516 (8.4)	525 (8.5)	0.005
Electrolyte Imbalance	3953 (19.6)	617 (9.9)	-0.28	576 (9.3)	617 (10)	0.02
Glaucoma /Cataract	6504 (32.3)	1782 (28.7)	-0.08	1775 (28.8)	1766 (28.6)	-0.003
HONK	204 (1)	44 (0.7)	-0.03	40 (0.7)	44 (0.7)	0.01
HTN nephropathy	1401 (7)	379 (6.1)	-0.03	354 (5.7)	377 (6.1)	0.02
Hyperthyroid disease	658 (3.3)	176 (2.8)	-0.03	170 (2.8)	175 (2.8)	0.005
Hypothyroid disease	2261 (11.2)	696 (11.2)	-0.001	722 (11.7)	685 (11.1)	-0.02
Osteomyelitis	272 (1.4)	38 (0.6)	-0.08	32 (0.5)	38 (0.6)	0.01
Pneumonia	2239 (11.1)	459 (7.4)	-0.13	493 (8)	457 (7.4)	-0.02
Skin infection	1096 (5.4)	295 (4.8)	-0.03	296 (4.8)	290 (4.7)	-0.005
Glucose-lowering therapy						
Metformin	13681 (67.9)	4788 (77.1)	0.2	4731 (76.7)	4752 (77)	0.008
Insulins	5973 (29.7)	1644 (26.5)	-0.07	1575 (25.5)	1620 (26.3)	0.02
SUs	10409 (51.7)	3205 (51.6)	-0.02	3154 (51.1)	3178 (51.5)	0.01
Glitazones	2576 (12.8)	962 (15.5)	0.08	978 (15.9)	954 (15.5)	-0.01
GLP-1 agonists	92 (0.5)	87 (1.4)	0.1	65 (1.1)	78 (1.3)	0.02
AGIs	988 (4.9)	196 (3.2)	-0.09	225 (3.7)	195 (3.2)	-0.03
Meglitinides	422 (2.1)	118 (1.9)	-0.01	98 (1.6)	113 (1.8)	0.02
Co-medications						
Anticoagulants	9958 (49.5)	2838 (45.7)	-0.1	2837 (46)	2825 (45.8)	0.01
Antiplatelets	9682 (48.1)	2781 (44.8)	-0.07	2783 (45.1)	2768 (44.9)	-0.05
Heparins	841 (4.2)	184 (3)	-0.07	190 (3.1)	182 (3)	-0.01
Thrombolytics	79 (0.4)	3 (0.1)	-0.07	3 (0.1)	3 (0.1)	0.01
Statins	13847 (68.8)	4813 (77.5)	0.2	4750 (77)	4775 (77.4)	0.01
Other lipid Lowerings	2704 (13.4)	1178 (19)	0.2	1129 (18.3)	1165 (18.9)	0.01
Nitrates	1808 (9.0)	446 (7.2)	-0.07	451 (7.3)	443 (7.2)	-0.005
Digoxin	1609 (8.0)	394 (6.3)	-0.06	392 (6.4)	391 (6.3)	-0.001
ACEIs	898 (4.5)	254 (4.1)	-0.02	255 (4.1)	252 (4.1)	-0.002
ARBs	11807 (58.6)	3749 (60.4)	0.01	3752 (60.8)	3723 (60.3)	-0.01
Entresto	3 (0)	2 (0)	0.05	0 (0)	2 (0)	0.03
Other Anti HTNs	11046 (54.9)	3014 (48.5)	-0.1	3009 (48.8)	2995 (48.5)	-0.005
Loop diuretics	3706 (18.4)	637 (10.3)	-0.2	638 (10.3)	632 (10.2)	-0.003
Other diuretics	4978 (24.7)	1478 (23.8)	-0.02	1509 (24.5)	1469 (23.8)	-0.02
Antianxieties	8676 (43.1)	2190 (35.3)	-0.2	2131 (34.5)	2176 (35.3)	0.02
Antipsychotics	1162 (5.8)	168 (2.7)	-0.2	163 (2.6)	168 (2.7)	0.005
Antidepressants	3711 (18.4)	898 (14.5)	-0.1	891 (14.4)	893 (14.5)	0.001
Dementia	2626 (13)	527 (8.5)	-0.1	516 (8.4)	525 (8.5)	0.005

Antiparkinsons	619 (3.1)	117 (1.9)	-0.08	114 (1.9)	117 (1.9)	0.004
Anticonvulsants	491 (2.4)	90 (1.5)	-0.07	90 (1.5)	90 (1.5)	0
NSAIDs	15558 (77.3)	4812 (77.5)	0.005	4828 (78.3)	4779 (77.5)	-0.02
Bisphos -phonates	858 (4.3)	156 (2.5)	-0.1	140 (2.3)	156 (2.5)	0.02
Opioids	9918 (49.3)	2656 (42.8)	-0.1	2610 (42.3)	2639 (42.8)	0.01

Values are represented as mean ± standard deviation or number (%); ACEis, angiotensin-converting enzyme inhibitors; AGIs, α-glucosidase Inhibitors; ARBs, angiotensin II receptor blockers; CABG, coronary artery bypass graft; CAD, coronary artery disease; COPD, chronic obstructive pulmonary disease; CV, cardiovascular; DM, diabetes mellitus; HONK, hyperglycaemic hyperosmolar nonketotic coma; HTN, hypertensive; MI, myocardial infarction; NSAIDs, non-steroidal anti-inflammatory drugs; PAD, peripheral artery disease; PUD, peptic ulcer disease; STD, standardized difference; SUs, sulfonylureas; UC, ulcerative colitis;

Table S3. Baseline characteristics of patients with normal kidney function before and after propensity matching

Variables	Pre-Match Sitagliptin N=284,665	Pre-Match Empagliflozin N=73,325	STD	Post-Match Sitagliptin N=73,217	Post-Match Empagliflozin N=73,217	STD
Sex, male	167,073 (58.7)	41,003 (55.9)	-0.02	40,896 (55.9)	40,955 (55.9)	-0.001
Age, year	54.5 (13)	49.8 (12.5)	-0.4	49.9 (13.1)	49.8 (12.5)	-0.01
Insurance type						
Normal	270,362 (95)	70,076 (95.6)	0.03	69,960 (95.6)	69,975 (95.6)	0.002
Medicaid	13,453 (4.7)	3,106 (4.2)		3,117 (4.3)	3,099 (4.2)	
No charge	850 (0.3)	143 (0.2)		140 (0.2)	143 (0.2)	
Number of Inpatient visit	0.6 (1.9)	0.4 (1.3)	-0.1	0.4 (1.5)	0.4 (1.3)	-0.01
Number of outpatient visit	25.8 (27.7)	25.1 (25.5)	-0.03	25.2 (26.3)	25.1 (25.5)	-0.01
Index year						
2016	83,000 (29.2)	16,199 (22.1)	0.2	16,028 (21.9)	16,198 (22.1)	0.007
2017	141,130 (49.6)	38,370 (52.3)		38,571 (52.7)	38,324 (52.3)	
2018	60,533 (21.3)	18,756 (25.6)		18,618 (25.4)	18,695 (25.5)	
Charlson comorbidity index						
0	26,481 (9.3)	5,108 (7.0)	0.1	4,899 (6.7)	5,107 (7.0)	0.01
1	31,973 (11.2)	7,521 (10.3)		7,618 (10.4)	7,514 (10.3)	
2	48,840 (17.2)	12,691 (17.3)		12,659 (17.3)	12,680 (17.3)	
3	177,371 (62.3)	48,005 (65.5)		48,041 (65.6)	47,916 (65.4)	
CV risk factor						
CAD	61,937 (21.8)	18,047 (24.6)	0.07	18,087 (24.7)	18,004 (24.6)	-0.003
Multi vessel CAD	29,888 (10.5)	9,505 (13.0)	0.08	9,469 (12.9)	9,473 (12.9)	0.0002
MI	3,262 (1.2)	1,321 (1.8)	0.05	1,319 (1.8)	1,314 (1.8)	-0.0005
CABG	496 (0.2)	176 (0.2)	0.01	159 (0.2)	176 (0.2)	0.005
Stroke	9,386 (3.3)	1,737 (2.4)	-0.006	1,771 (2.4)	1,735 (2.4)	-0.003
PAD	4,919 (1.7)	1,274 (1.7)	0.0007	1,331 (1.8)	1,274 (1.7)	-0.006
DM circulation	23,177 (8.1)	7,648 (10.4)	0.08	7,644 (10.4)	7,623 (10.4)	-0.001
DM foot	26 (0.01)	10 (0.01)	0.004	8 (0.01)	9 (0.01)	0.001
DM nephropathy	-	-	-	-	-	-
DM neuropathy	29,804 (10.5)	8,884 (12.1)	0.05	9,009 (12.3)	8,856 (12.1)	-0.006
DM other Complications	204,338 (71.8)	53,566 (73.1)	0.03	53,920 (73.6)	53,480 (73.0)	-0.01
Hyperglycemia	4,087 (1.4)	919 (1.3)	-0.02	913 (1.3)	919 (1.3)	0.0007
Comorbidities						
Hypertension	161,970 (56.9)	44,394 (60.5)	0.07	44,604 (60.9)	44,306 (60.5)	-0.008
Edema	25,656 (9.0)	6,727 (9.2)	0.006	6,823 (9.3)	6,714 (9.2)	-0.005
Kidney stone	4,055 (1.4)	1,129 (1.5)	0.01	1,147 (1.6)	1,128 (1.5)	-0.002
Osteoarthritis	86,191 (30.3)	20,471 (27.9)	-0.05	20,691 (28.3)	20,448 (27.9)	-0.007
Other arthritis	64,949 (22.8)	15,887 (21.7)	-0.03	16,126 (22)	15,872 (21.7)	-0.008
PUD	66,005 (23.2)	16,779 (22.9)	-0.007	16,941 (23.1)	16,759 (22.9)	-0.006
Pancreatitis	4,093 (1.4)	1,014 (1.4)	-0.005	1,010 (1.4)	1,009 (1.4)	-0.0001
UC	434 (0.2)	98 (0.1)	-0.005	94 (0.1)	98 (0.1)	0.002
Crohn	114 (0.04)	20 (0.03)	-0.007	16 (0.02)	20 (0.03)	0.004

Asthma	37,306 (13.1)	9,649 (13.2)	0.002	9,682 (13.2)	9,634 (13.2)	-0.002
COPD	7,909 (2.8)	1,343 (1.8)	-0.06	1,379 (1.9)	1,342 (1.8)	-0.004
Bladder stone	195 (0.1)	36 (0.1)	-0.008	35 (0.1)	36 (0.1)	0.0006
Dementia	29,125 (10.2)	5,480 (7.5)	-0.1	5,542 (7.6)	5,478 (7.5)	-0.003
Electrolyte Imbalance	15,403 (5.4)	3,457 (4.7)	-0.03	3,436 (4.7)	3,451 (4.7)	0.001
Glaucoma /Cataract	64,771 (22.8)	15,888 (21.7)	-0.03	16,029 (21.9)	15,857 (21.7)	-0.006
HONK	1,613 (0.6)	373 (0.5)	-0.008	370 (0.5)	372 (0.5)	0.0004
HTN nephropathy	-	-	-	-	-	-
Hyperthyroid disease	5,679 (2)	1,575 (2.2)	0.01	1,594 (2.2)	1,570 (2.1)	-0.002
Hypothyroid disease	15,581 (5.5)	4,918 (6.7)	0.05	4,911 (6.7)	4,908 (6.7)	-0.0002
Osteomyelitis	2,006 (0.7)	365 (0.5)	-0.03	354 (0.5)	365 (0.5)	0.002
Pneumonia	20,378 (7.2)	4,604 (6.3)	-0.04	4,637 (6.3)	4,597 (6.3)	-0.002
Skin infection	1,2045 (4.2)	3,213 (4.4)	0.007	3,255 (4.5)	3,208 (4.4)	-0.003
Glucose-lowering therapy						
Metformin	177,686 (62.4)	48,848 (66.6)	0.09	48,963 (66.9)	48,756 (66.6)	-0.006
Insulins	36,597 (12.9)	10,158 (13.9)	0.03	10,093 (13.8)	10,110 (13.8)	0.0007
SUs	111,365 (39.1)	32,123 (43.8)	0.1	32,448 (44.3)	32,043 (43.8)	-0.01
Glitazones	20,763 (7.3)	7,887 (10.8)	0.1	7,997 (10.9)	7,852 (10.7)	-0.006
GLP-1 agonists	481 (0.2)	490 (0.7)	0.08	377 (0.5)	450 (0.6)	0.01
AGIs	9,103 (3.2)	1,898 (2.6)	-0.04	1,949 (2.7)	1,896 (2.6)	-0.005
Meglitinides	1,205 (0.4)	298 (0.4)	-0.003	316 (0.4)	298 (0.4)	-0.004
Co-medications						
Anticoagulants	96,281 (33.8)	27,394 (37.4)	0.07	27,486 (37.5)	27,338 (37.3)	-0.004
Antiplatelets	93,509 (32.9)	26,625 (36.3)	0.07	26,839 (36.7)	26,571 (36.3)	-0.008
Heparins	5,407 (1.9)	1,313 (1.8)	-0.008	1,304 (1.8)	1,310 (1.8)	0.0006
Thrombolytics	256 (0.1)	25 (0.03)	-0.02	19 (0.03)	25 (0.03)	0.005
Statins	142,130 (49.9)	45,578 (62.2)	0.2	45,648 (62.4)	45,478 (62.1)	-0.005
Other lipid Lowerings	28,350 (10.0)	10,508 (14.3)	0.1	10,417 (14.2)	10,467 (14.3)	0.002
Nitrates	14,790 (5.2)	4,815 (6.6)	0.06	4,850 (6.6)	4,796 (6.6)	-0.003
Digoxin	13,182 (4.6)	4,326 (5.9)	0.06	4,360 (6)	4,309 (5.9)	-0.003
ACEIs	6,375 (2.2)	2,228 (3.0)	0.05	2,272 (3.1)	2,212 (3.0)	-0.005
ARBs	122,463 (43.0)	36,242 (49.4)	0.1	36,418 (49.7)	36,159 (49.4)	-0.007
Entresto	14 (0.01)	32 (0.04)	0.02	12 (0.02)	25 (0.03)	0.01
Other Anti HTNs	123,988 (43.6)	33,223 (45.3)	0.04	33,349 (45.6)	33,149 (45.3)	-0.006
Loop diuretics	19,711 (6.9)	4,613 (6.3)	-0.03	4,635 (6.3)	4,596 (6.3)	-0.002
Other diuretics	58,239 (20.5)	15,990 (21.8)	0.03	16,112 (22)	15,952 (21.8)	-0.006
Antianxieties	103,758 (36.5)	24,669 (33.6)	-0.06	24,744 (33.8)	24,634 (33.7)	-0.003
Antipsychotics	10,823 (3.8)	1,977 (2.7)	-0.06	2,083 (2.8)	1,976 (2.7)	-0.009
Antidepressants	38,511 (13.5)	9,174 (12.5)	-0.03	9,301 (12.7)	9,159 (12.5)	-0.01
Dementia	29,125 (10.2)	5,480 (7.5)	-0.1	5,542 (7.6)	5,478 (7.5)	-0.003
Antiparkinsons	6,034 (2.1)	1,083 (1.5)	-0.05	1,119 (1.5)	1,081 (1.5)	-0.004

Anticonvulsants	5,421 (1.9)	1,133 (1.6)	-0.03	1,127 (1.5)	1,130 (1.5)	0.0003
NSAIDs	216,954 (76.2)	56,826 (77.5)	0.03	57,157 (78.1)	56,748 (77.5)	-0.01
Bisphos-phonates	10,443 (3.7)	1,774 (2.4)	-0.07	1,785 (2.4)	1,773 (2.4)	-0.001
Opioids	123,349 (43.3)	30,499 (41.6)	-0.04	30,653 (41.9)	30,459 (41.6)	-0.005

Values are represented as mean ± standard deviation or number (%); ACEis, angiotensin-converting enzyme inhibitors; AGIs, α-glucosidase Inhibitors; ARBs, angiotensin II receptor blockers; CABG, coronary artery bypass graft; CAD, coronary artery disease; COPD, chronic obstructive pulmonary disease; CV, cardiovascular; DM, diabetes mellitus; HONK, hyperglycaemic hyperosmolar nonketotic coma; HTN, hypertensive; MI, myocardial infarction; NSAIDs, non-steroidal anti-inflammatory drugs; PAD, peripheral artery disease; PUD, peptic ulcer disease; STD, standardized difference; SUs, sulfonylureas; UC, ulcerative colitis;

Table S4. Sensitivity analysis

	Adjusted hazard ratio (95% CI)		
**Outcomes**	**Original analysis**	**Sensitivity analysis I**	**Sensitivity analysis II**
MACEs	0.74 (0.64, 0.85)	0.78 (0.62, 0.97)	0.73 (0.63, 0.86)
All-cause death	0.47 (0.33, 0.68)	0.66 (0.44, 0.97)	0.76 (0.59, 0.97)
Myocardial infarction	0.60 (0.45, 0.81)	0.45 (0.34, 0.60)	0.41 (0.33, 0.52)
Stroke	0.92 (0.74, 1.13)	0.99 (0.72, 1.34)	1.02 (0.79, 1.32)
Hospitalization for unstable angina	0.67 (0.57, 0.79)	0.68 (0.55, 0.84)	0.72 (0.60, 0.85)
Coronary revascularization	0.79 (0.65, 0.97)	0.76 (0.61, 0.96)	0.79 (0.66, 0.96)
Transient ischemic attack	0.92 (0.72, 1.17)	0.89 (0.69, 1.14)	0.92 (0.72, 1.18)
Hospitalization for heart failure	0.66 (0.55, 0.80)	0.65 (0.50, 0.83)	0.68 (0.56, 0.84)
Hypoglycemic adverse event	0.78 (0.62, 0.97)	0.73 (0.54, 0.98)	0.75 (0.59, 0.94)
Urinary tract infection	0.90 (0.82, 0.98)	0.84 (0.76, 0.93)	0.90 (0.83, 0.98)
Genital infection	1.43 (1.27, 1.61)	1.57 (1.37, 1.80)	1.45 (1.29, 1.63)
Acute kidney injury	0.94 (0.76, 1.17)	0.77 (0.57, 1.03)	0.95 (0.75, 1.20)
Volume depletion	0.90 (0.78, 1.04)	0.94 (0.79, 1.12)	0.95 (0.82, 1.11)
Diabetic ketoacidosis	0.96 (0.60, 1.54)	0.86 (0.49, 1.50)	1.05 (0.63, 1.75)
Thromboembolic event	0.88 (0.73, 1.05)	0.91 (0.73, 1.13)	0.91 (0.75, 1.09)
Fracture	1.03 (0.92, 1.14)	1.13 (0.99, 1.29)	1.09 (0.97, 1.22)
